# Comparative Transcriptome Analysis Points to the Biological Processes of Hybrid Incompatibility between *Brassica napus* and *B. oleracea*

**DOI:** 10.3390/plants12142622

**Published:** 2023-07-12

**Authors:** Fang Yue, Fajing Zheng, Qinfei Li, Jiaqin Mei, Chunlei Shu, Wei Qian

**Affiliations:** 1 College of Agronomy and Biotechnology, Southwest University, Chongqing 400715, China; 2 Academy of Agricultural Sciences, Southwest University, Chongqing 400715, China; 3 College of Horticulture and Landscape, Southwest University, Chongqing 400715, China

**Keywords:** *Brassica napus*, *Brassica oleracea*, *Brassica rapa*, hybrid incompatibility, ROS, RNA-seq

## Abstract

Improving *Brassica napus* via introgression of the genome components from its parental species, *B. oleracea* and *B. rapa*, is an important breeding strategy. Interspecific hybridization between *B. napus* and *B. rapa* is compatible with high rate of survival ovules, while the hybridization between *B. napus* and *B. oleracea* is incompatible with the high occurrence of embryo abortion. To understand the diverse embryo fate in the two interspecific hybridizations, here, the siliques of *B. napus* pollinated with *B. oleracea* (AE) and *B. rapa* (NE) were employed for transcriptome sequencing at 8 and 16 days after pollination. Compared to NE and the parental line of *B. napus*, more specific differentially expressed genes (DEGs) (1274 and 1698) were obtained in AE and the parental line of *B. napus* at 8 and 16 days after pollination (DAP). These numbers were 51 and 5.8 times higher than the number of specific DEGs in NE and parental line of *B. napus* at 8 and 16 DAP, respectively, suggesting more complex transcriptional changes in AE. Most of DEGs in the terms of cell growth and cell wall formation exhibited down-regulated expression patterns (96(down)/131(all) in AE8, 174(down)/235(all) in AE16), while most of DEGs in the processes of photosynthesis, photorespiration, peroxisome, oxidative stress, and systemic acquired resistance exhibited up-regulated expression patterns (222(up)/304(all) in AE8, 214(up)/287(all) in AE16). This is in accordance with a high level of reactive oxygen species (ROS) in the siliques of *B. napus* pollinated with *B. oleracea*. Our data suggest that the disorder of plant hormone metabolism, retardation of cell morphogenesis, and the accumulation of ROS may be associated with hybrid incompatibility between *B. napus* and *B. oleracea*.

## 1. Introduction

Rapeseed (*Brassica napus* L.) is a globally significant oil crop, ranking second only to soybean in terms of production. It is cultivated across approximately 35 million hectares of land in China, Europe, Canada, and Australia [1], with annual production of 70 million tons of oilseed (accessed on 1 January 2023 http://www.fao.org/faostat/en/#data/QC/visualize). The circular economy benefits from rapeseed due to its abundant supply of vegetable oil and protein-rich meal [2]. Originating from Europe through spontaneous hybridization between *B. rapa* (2*n* = 20, AA) and *B. oleracea* (2*n* = 18, CC) [3], rapeseed has a short cultivation history. However, extensive breeding efforts aimed at enhancing rapeseed quality, specifically by reducing its erucic acid and glucosinolate content (double-low traits), have led to a narrowing of the genetic diversity within current rapeseed varieties [4,5].

In contrast, the two diploid ancestor species of *B. napus* exhibit broad genetic variation and have been extensively utilized to expand the genetic diversity of rapeseed [6,7,8]. *B. rapa*, with two independent centers of origin in East Asia and Europe [9], displays significant morphological and agronomic variability. The introgression of genomic components from East Asian *B. rapa* has played a pivotal role in expanding the genetic diversity of rapeseed [6,9,10]. Similarly, the genus *B. oleracea* encompasses various cultivated forms and at least ten wild taxa distributed throughout the Mediterranean and eastern Atlantic coastal areas [11]. Among these, the cultivated forms and five wild taxa of *B. oleracea* (*B. incana*, *B. bourgeaui*, *B. montana*, *B. oleracea* ssp. *oleracea* and *B. cretica*) share a closer relationship with the C subgenome of rapeseed. On the other hand, the other five wild taxa of *B. oleracea* (*B. macrocarpa*, *B. rupestris*, *B. villosa*, *B. insularis* and *B. hilarionis*) exhibit a greater genetic distance from the C subgenome of *B. napus*, presenting an opportunity to effectively broaden the diversity of the C subgenome in rapeseed [12]. The introgression of parental species has been successfully employed to transfer desirable traits into current rapeseed, including multilocular rapeseed [13], yellow-seeded rapeseed [14,15], prematurity [16,17], self-incompatibility [18], and resistance to disease [19,20]. Furthermore, the introgression of parental species enhances the potential of heterosis in rapeseed [6,21].

It is feasible to develop interspecific hybridization between *B. napus* and *B. rapa*, while embryo abortion occurs significantly in the hybridization between *B. napus* and *B. oleracea* [22]. However, the underlying molecular mechanism governing the divergent fate of embryos in these two hybridizations remains largely unknown. Therefore, this study aims to elucidate a comparative transcriptomic analysis of rapeseed siliques pollinated with B. oleracea and B. rapa. Through the identification of differentially expressed genes, we focus on processes such as cell elongation, plant hormone regulation, reactive oxygen species (ROS) production, and stress responses, which may be associated with embryo abortion in rapeseed siliques pollinated with B. oleracea.

## 2. Results

**Figure 1:**
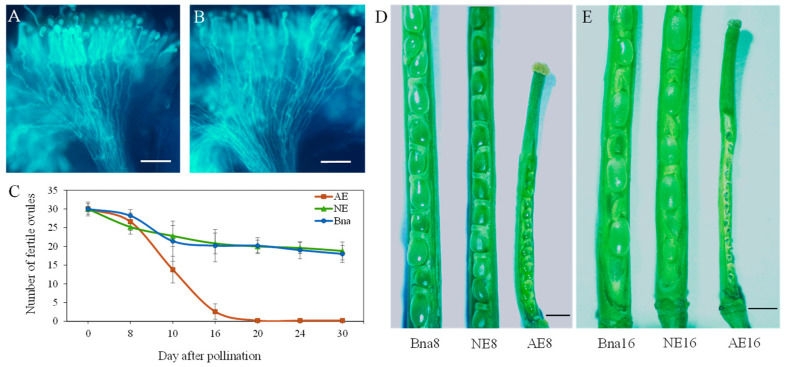
Pollen–stigma reaction and ovule development in the silique from *B. napus* pollinated with *B. oleracea* and *B. rapa*. (**A**,**B**): Stigma recognition responses on *B. napus* stigma pollinated with *B. oleracea* (**A**) and *B. rapa* (**B**) at 4 h after pollination. Bars = 100 μm. (**C**) The number of developed ovules of *B. napus* pollinated with *B. oleracea* (AE) and *B. rapa* (NE) and in parental line of *B. napus* (Bna) during embryo development. (**D**,**E**) The ovules’ morphology in the silique from *B. napus* pollinated with *B. oleracea*, *B. rapa* and parental line of *B. napus* at 8 (**D**) and 16 (**E**) days after pollination. Bars = 5 mm.

In accordance with previous studies [23], pollen grains from *B. oleracea* (M245) and *B. rapa* (M240) are able to germinate on the stigma of *B. napus* (Zhongshuang 11) and penetrate through the stigma at 4 h after pollination (Figure 1A,B), suggesting a normal pollen–stigma recognition reaction in both two interspecies hybridizations. However, differences in ovule development after pollination were detected in two hybridizations. The silique and ovules from *B. napus* pollinated with *B. rapa* exhibited the same length and size as those of parental line of *B. napus*, but the silique and ovules from *B. napus* pollinated with *B. oleracea* were shorter and smaller at 8 and 16 days after pollination (DAP) (Figure 1D,E). In spite of no significant difference in the number of the enlarged ovules between the two hybridizations at 8 DAP, with an average of 28 ovules per silique (Figure 1C,D), almost all ovules pollinated with *B. oleracea* shrank and died at 16 DAP, while the ovules pollinated with *B. rapa* developed as alongside the parental line of *B. napus* (Figure 1E).

Considering that the silique wall is one of the main photosynthetic organs after flowering, and is the site of seed formation in rapeseed, we collected rapeseed siliques pollinated with *B. oleracea* (AE) and *B. rapa* (NE) together with parental line of *B. napus* (Bna) for transcriptome sequencing at 8 and 16 DAP. Six sets of cDNA libraries (AE8, AE16, NE8, NE16, Bna8 and Bna16) were sequenced on the Illumina Hiseq 2000^TM^ platform, producing more than 30 Gb clean reads. Average of 72.3% reads were precisely mapped to the *B*. *napus* reference genome (accessed on 10 July 2021 https://www.ncbi.nlm.nih.gov/genome/?term=brassica+napus).

**Figure 2:**
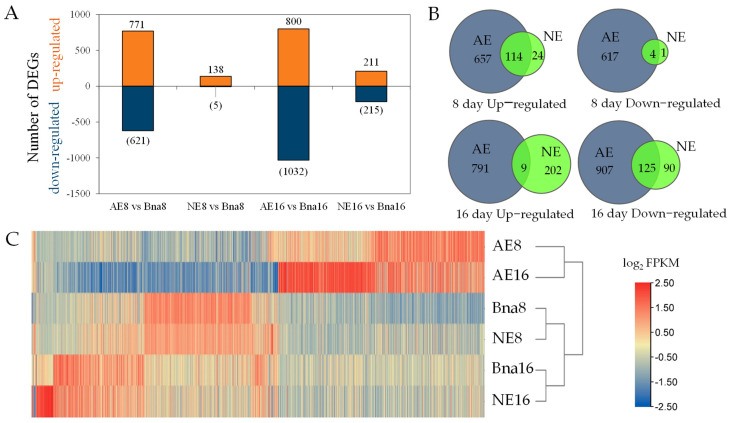
Differentially expressed genes (DEGs) in rapeseed silique pollinated with *B. oleracea* (AE) and *B. rapa* (NE) compared to the rapeseed parental line of *B. napus* (Bna) at 8 and 16 days after pollination (DAP). (**A**) The number of up- or down-regulated DEGs in AE and NE at 8 and 16 DAP. (**B**) Venn diagram of DEGs in AE and NE at 8 and 16 DAP. (**C**) Heatmap of the expression pattern of DEGs in silique from parental line of *B. napus*, AE and NE at 8 and 16 DAP.

In comparison with parental line of *B. napus* at 8 and 16 DAP, there were 1392 and 1832 differentially expressed genes (DEGs) in the silique pollinated with *B. oleracea*, and 143 and 426 DEGs in the silique pollinated with *B. rapa* (Figure 2A, Table S2), with the overlap of 118 and 134 DEGs (Figure 2B), respectively. To verify the DEGs identified using RNA-seq data, twenty DEGs of interest were employed for qRT-PCR. We found that the expression tendency of those genes via qRT-PCR was in general agreement with the results of RNA-seq (Figure S1). The heatmap of the expression level of DEGs in silique revealed that the parental line of *B. napus* exhibited a similar expression pattern to the hybridization between *B. napus* and *B. rapa*, but one distant from the hybridization between *B. napus* and *B. oleracea* (Figure 2C), indicating more changes to the transcriptome in the silique of hybridization between *B. napus* and *B. oleracea*.

**Figure 3:**
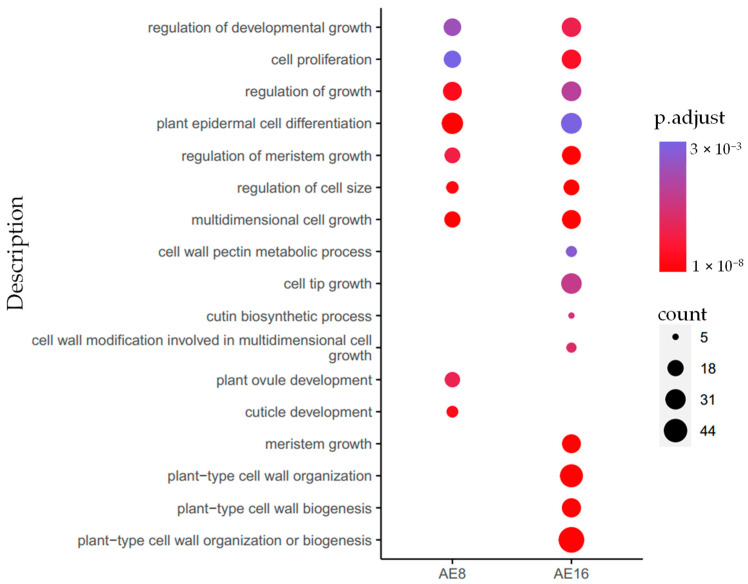
GO terms enriched with the down-regulated genes related to cell growth in the silique pollinated with *B. oleracea* (AE) at 8 and 16 days after pollination compared to parental line of *B. napus*. Each dot represents one GO gene set, where the size indicates the enriched genes’ number, and the color intensity is proportional to the enrichment significance (*p.adjust*). The full list of significantly enriched GO terms for DEGs is given in Table S3.

We speculate that changes in gene expression may be associated with the diverse fate of ovule development in two hybridizations. To verify this speculation, we filtered the overlapped DEGs between AE and NE, and gained 25 and 292 specific DEGs in NE at 8 and 16 DAP, respectively. However, more specific DEGs (1274 and 1698) were obtained in AE at 8 and 16 DAP, which were enriched into 67 and 65 GO terms of biological processes (Table S3). Of these, 9 and 15 GO terms related to cell growth in AE8 and AE16 and harbored a high ratio of down-regulated DEGs (96(down)/131(all) in AE8, 174(down)/235(all) in AE16), with 7 overlapping terms: “multidimensional cell growth”, “plant epidermal cell differentiation”, “regulation of cell size”, “regulation of growth”, “regulation of meristem growth”, “regulation of developmental growth”, and “cell proliferation” (Figure 3). There were 64 down-regulated DEG-encoded cell well components in AE, including 10 xyloglucan endotransglucosylase/hydrolases (XTHs), 7 expansins (EXPs), 7 extensins (EXTs) and 4 polygalacturonases (PGases), 5 glycoside hydrolases (GH), and 5 arabinogalactan proteins (AGPs), 13 epidermis cells, and 13 cytoskeleton structures (Figure S2). *XTHs* contribute to cell expansion and secondary wall formation [24,25]. We found homologous genes of *AtXTH4* (*LOC106389424*, *LOC106439779*) and *AtXTH20* (*LOC106385619*, *LOC106408546*) were significant declined 2.20~53.66-fold in AE, but there was no significant expression change in NE (Figure S2). *EXT3* forms covalently cross-linked networks in primary cell walls, the knockout mutation of which results in a cell lethal phenotype in *Arabidopsis* [26]. We found three homologous genes of *AtEXT3* (*LOC106352980*, *LOC106358201*, *LOC106435427*) were down-regulated 2.71~17.22-fold in AE, but there was no significant expression change in NE, except of *LOC106435427*, which was up-regulated 3.17-fold in NE16. *AtSTK* affects fertilization and seed formation by regulating the formation of the maternal endothelium [27,28]. In comparison with the parental line of *B. napus*, three homologous genes of *STK* (*LOC106439143*, *LOC106360950* and *LOC106393661*) were down-regulated in their expression by 3.61~11.81-fold in AE; there was no significant change in NE.

Auxin and cytokinin play central roles in plant growth and development by controlling cell division, elongation, and differentiation [29]. We found 80 down-regulated genes involved in the biological processes of auxin synthesis, indoleacetic acid biosynthetis, auxin polar transport, the response to cytokinin, and cytokinin-activated signaling pathways in AE (Figure S2). For example, the homologous genes of *AtABCB1* (*LOC106391495*), *AtTAR2* (*LOC106375778*) and *AtAHK* (*LOC106431570* and *LOC106410863*), which promote cell division and elongation by mediating auxin and cytokinin signals [30,31,32], were down-regulated 2.65~14.6-fold in AE8, but there were no significant changes in NE8. These results are in accordance with the observation of abortive embryos in AE.

Among the top 25 GO terms enriched by DEGs in AE8, there were 5 GO terms harboring 146 up-regulated and 38 down-regulated genes related to photosynthesis, including “photosynthesis”, “oxidoreduction coenzyme metabolic process”, “response to red light”, “photosystem II assembly” and “photosynthetic electron transport in PSI” (Table S3). KEGG analysis also showed that six photosynthesis-related pathways were significantly enriched, including “carbon metabolism”, “glyoxylate and dicarboxylate metabolism glyoxylic acid”, “carbon fixation in photosynthetic organisms”, “peroxisomes”, “photosynthetic antenna proteins” and “photosynthesis” (Table S4). A similar observation of high ratio of up-regulated DEGs was found in AE16, where 154 up-regulated and 37 down-regulated DEGs were enriched in 4 GO terms related to photosynthesis, with 84 DEGs overlapping with AE8 (Table S2). This indicated stronger photosynthesis in AE.

**Figure 4:**
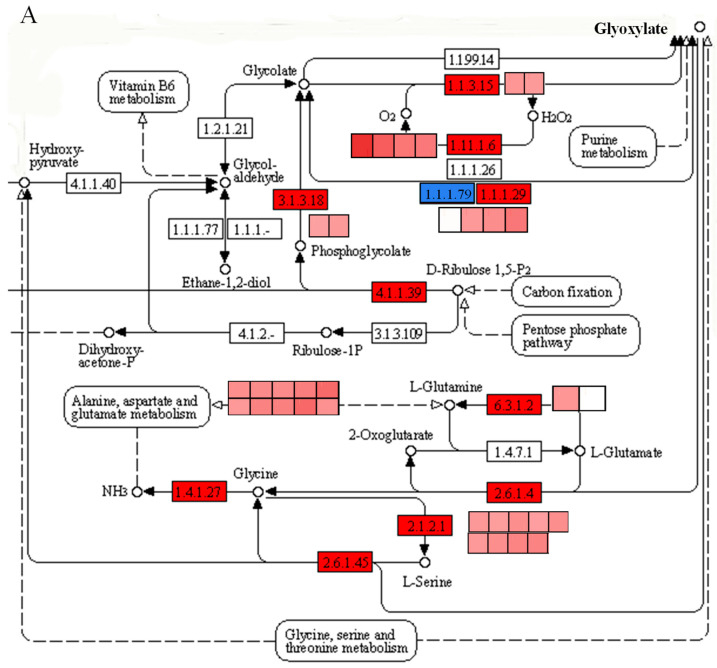

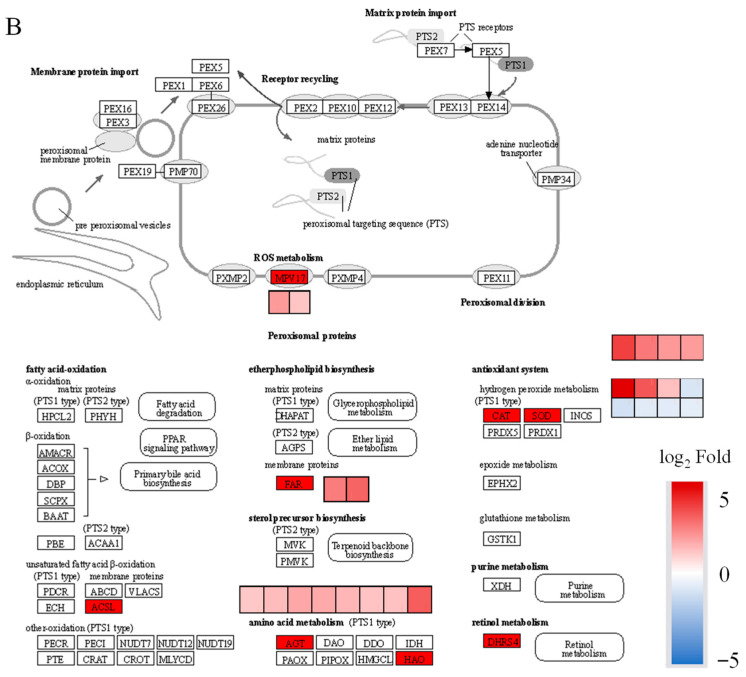
DEGs in the KEGG pathways of glyoxylate and dicarboxylate metabolism (**A**), and peroxidase (**B**) in AE8, compared to the parental line of *B. napus*. The grids of red and blue in the metabolic pathway indicate the enrichment of up-regulated and down-regulated DEGs, and the adjacent squares indicate the differentially expression genes. The color in the square represents expression changes based on the log_2_ fold. The full list of significantly enriched KEGG pathways for DEGs is given in Table S4.

Photorespiration is a side reaction of photosynthesis which produces hydrogen peroxide through glycolate pathway [33]. We found that 62 DEGs were significantly enriched in the KEGG pathways of glyoxalic acid and diacetic acid metabolism and peroxisome in AE8 (Figure 4A,B). Of these, 29 genes were continuously up-regulated in expression in AE16. For example, four homologous genes of *AGT1* (*LOC106389407*, *LOC106439758*, *LOC106410465*, *LOC106396365*) encoded peroxisome in photorespiration, and were up-regulated in expression 2.31 to 3.38-fold in AE; however, there was no significant expression change in NE. *AtSHM1* encoded serine hydroxymetyltransferase, which is involved in the photorespiratory pathway of dissipation mechanisms, and the mutation of which results in increased oxidative stress [34]. We found that two homologous genes of *AtSHM1* (*LOC106361388* and *LOC106436527*) were up-regulated in expression 2.08 and 2.77-fold in AE8, but declined 1.14 and 1.01-fold in NE8.

**Figure 5:**
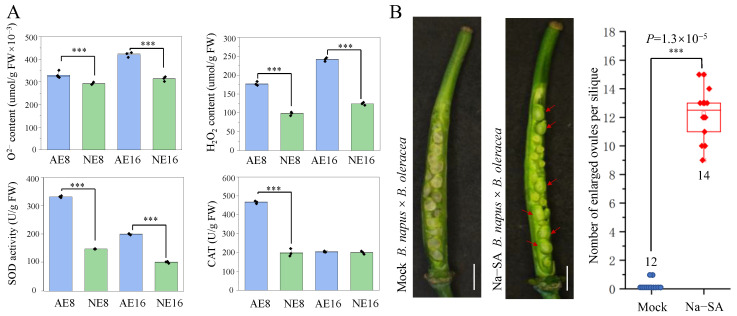
High reactive oxygen species (ROS) level blocks the ovule development in the silique of *B. napus* pollinated with *B. oleracea*. (**A**) Determination of the content of O^2−^ and H_2_O_2_ and enzyme activity of superoxide dismutase (SOD) and catalase (CAT) in rapeseed silique crossed with *B. oleracea* (AE) and *B. rapa* (NE) at 8 and 16 DAP. (**B**) Developing ovules and the number of the enlarged ovules in *B. napus* × *B. oleracea* via spraying 5 mmol Na-SA in pistil prior to pollination (Na-SA) and spraying water at 10 DAP (Mock). The red arrowheads indicate enlarged ovules. Bar = 1 mm. *p*-values were determined via two-tailed Student’s *t*-tests. *** Significant difference at *p* < 0.001.

The acumination of ROS production promotes the up-regulated expression of genes in the ROS scavenging system [35]. We found that homologous genes of *CAT2* (*LOC106436083* and *LOC106421785*) and *CAT3* (*LOC106358221* and *LOC106431162*) encoded by peroxisomal catalase were up-regulated in expression 3.93~13.07-fold in AE8, and 2.16~3.92-fold in AE16; however, there was no significant expression change in NE (Figure 4B). Three homologous genes of *AtFSD1* (*LOC106346563*, *LOC106442544* and *LOC106411335*) encoded by superoxide dismutase (SOD) were up-regulated in expression 2.45, 9.92 and 31.6-fold in AE8, but were down-regulated 1.02~1.10-fold in NE8, except for *LOC106411335*, which saw a 1.97-fold increase (Figure 4B). To verify the differences in ROS production and scavenging in siliques between AE and NE, we assayed the content of O^2−^ and H_2_O_2_ and the enzyme activity of SOD and CAT in the silique, ovules, and pericarp at 8 and 16 DAP (Figure S3). The contents of O^2−^ and H_2_O_2_ accumulated in AE were 1.19~1.81 times higher than those in NE at 8 and 16 DAP, while the enzyme activities of SOD and CAT in AE were 2.34~2.95 times higher than those in NE at 8 and 16 DAP (Figure 5A). Considering that the success of interspecific crosses in *Brassica* can be increased by spraying sodium salicylate (Na-SA) to reduce the level of ROS [36], we treated *B. napus* pistils with Na-SA prior to pollination with *B. oleracea*, and found more enlarged ovules in the silique at 10 DAP (Figure 5B,C), suggesting that a high level of ROS inhibits ovule development in AE.

Excessive ROS can launch a series of stress responses in cells [37]. We found 7 GO terms and 4 KEGG pathways related to stress response in AE (Tables S3 and S4), harboring 169 DEGs. The homologs of *KTI1* (*LOC106430798*), *ESP* (*LOC106348445*, *LOC106415866*, *LOC106345821*), *RD21* (*LOC106422572*, *LOC106454047*), *DRT112* (*LOC106433715*), *WRKY70* (*LOC106367975*) and *HSPRO2* (*LOC106450828*) involved in plant defense and hypersensitivity reactions [38,39,40,41,42,43] were up-regulated in expression 3.73~104.1-fold in AE, but there was no significant expression change in NE (Table S2). Among 9 DEGs related to ABA as a stress hormone in the KEGG pathway of plant hormone signal transduction, 8 genes were up-regulated in expression in AE8 (Table S4). These findings indicated that the silique pollinated with *B. oleracea* was under significant stress during development.

## 3. Discussion

Hybridization between rapeseed and its parental species, *B. oleracea* and *B. rapa*, is a valuable strategy for introducing parental genome components into rapeseed. However, hybrid incompatibility poses a significant barrier to the introgression of the *B. oleracea* genome [22,44]. In this study, we observed a higher number of differentially expressed genes (DEGs) in rapeseed siliques pollinated with *B. oleracea* compared to those pollinated with *B. rapa*, when compared to rapeseed without pollination. Notably, the down-regulated DEGs were enriched in the biological processes related to cell elongation and cell wall formation (auxin and cytokinin). On the other hand, the up-regulated DEGs were enriched in processes associated with photosynthesis and stress responses in rapeseed siliques pollinated with *B. oleracea*. These findings suggest that disrupted plant hormone metabolism, retardation of cell morphogenesis, and accumulation of reactive oxygen species (ROS) may be associated with embryo abortion in rapeseed siliques pollinated with *B. oleracea*. These insights provide valuable information on distant hybrid incompatibility in the *Brassica* genus.

Photorespiration is essential for plant survival under high-intensity photosynthesis, and prevents photoinhibition by accumulating the by-products of ROS [45]. Further investigations revealed more up-regulated DEGs involved in photorespiration and peroxisomes, as well as higher levels of O^2−^ and H_2_O_2_ and the increased enzyme activity of superoxide dismutase (SOD) and catalase (CAT) in siliques with abnormal embryos (AE) compared to siliques with normal embryos (NE). Although we did not verify that the activities of photorespiration and peroxisomes are directly related to the production of ROS in AE, up-regulation of the genes involved in those processes may function as a mediator of active O^2−^ and H_2_O_2_ generation, and may strongly induce the activities of SOD and CAT in siliques of AE. In addition, treating AE siliques with a scavenger of ROS resulted in the observation of more enlarged ovules. These findings indicate that the embryos in AE are subjected to significant oxidative stress during ovules’ development. Consistent with these observations, we detected strong stress signals in AE, with 64 up-regulated DEGs associated with various stress responses. Apetala 2/ethylene-responsive factor (AP2/ERF) proteins are known for their roles in plant development and stress resistance [46,47]. Genetic studies have shown that genes encoding specific AP2/ERF proteins are pivotal in plant embryo development and seed germination [48]. Knockdown of *CmERF12*, which encodes a specific AP2/ERF protein, has been shown to promote embryo development and increase seed setting rates in chrysanthemum’s distant hybridization [49]. In this study, we identified a similar AP2/ERF domain in *LOC106390866*, which was up-regulated expression by 3.70 times in AE8, but showed no significant expression change in NE8 when compared to the parental line of *B. napus*. This suggests that *LOC106390866* may play same role as *CmERF12* in the distant hybridization incompatibility between *B. napus* and *B. oleracea*.

Excessive ROS can damage cellular components and destroy cell integrity [50], causing an imbalance in phytohormones [51]. In this study, we observed significant down-regulation of genes associated with auxin and cytokinin signaling in AE. For instance, genes such as *ABCB1*, *XTH4* and *GASA1,* which are involved in cell wall components, cell elongation, and auxin biosynthesis, were significantly down-regulated in expression in AE [52,53,54] (Table S2). This finding suggests a link between growth hormones and embryo abortion. Previous studies have shown that the content of auxin and cytokinin in aborted embryos is significantly lower than in normal embryos in Chinese white poplar [55]. Additionally, transient application of N-1-naphthylphthalamic acid (NPA), an auxin inhibitor, on *Arabidopsis* gynoecium leads to ovule abortion [56]. Similarly, hybrid embryos between rapeseed and *B. oleracea* can be rescued using culture medium supplemented with growth hormone (kinetin, naphthaleneacetic acid) in a practical breeding program [18].

## 4. Materials and Methods

### 4.1. Plant Materials

The *B. napus* cultivar “Zhongshuang 11”, *B. oleracea* inbred line “M245” and *B. rapa* inbred line “M240” were grown in the experimental field of Southwest University, Chongqing, China. The stamens of “Zhongshuang 11” were emasculated and pollinated with the fresh pollen of *B. oleracea* and *B. rapa*, to develop hybrids between *B. napus* and *B. oleracea* and between *B. napus* and *B. rapa,* three times, with an interval of 5 days, producing at least 100 hybrid siliques during each interval.

### 4.2. Morphological Observation

Stigma recognition response. To observe the pollen–stigma recognition response, the pistils were collected 4 h after pollination and fixed in Carnoy’s solution (Vethanol:Vacetic acid = 3:1) at 4 °C for 24 h. Pistils were then treated with 8 mol/l NaOH for 8 h at room temperature, and stained with 0.1% aniline blue solution before observation with a fluorescence microscope [57].

Ovule number. The initial ovules were counted using the method of unpollinated stigmas of *B. napus* treated with transparent agent. The enlarged embryos of self-pollinated *B. napus* and the hybrid embryos of *B. napus* with *B. oleracea* and *B. napus* with *B. oleracea* were counted at 8, 10, 16, 20, 24, 30 days, together with the initial ovules prior to pollination.

### 4.3. Transcriptome Sequencing and Differentially Expressed Gene Analysis

The siliques from *B. napus* cv. “Zhongshuang 11” pollinated with *B. oleracea* and *B. rapa* were collected together with those of the parental line of *B. napus* at 8 and 16 days after pollination, in triplicate, and bulked to produce six sets of samples. The mRNA of samples was extracted using an Eastep^®^ Supper kit (Promega, Shanghai, China) and sequenced on the Illumina Hiseq 2000^TM^ platform from Gene Denove Technologies (Guangzhou, China), producing six sets of transcriptome data: two sets from the silique pollinated with *B. oleracea* (AE8 and AE16), two sets from the silique pollinated with *B. rapa* (NE8 and NE16), and two sets from parental line of *B. napus* (Bna8 and Bna16).

The raw sequencing reads were mapped to the rapeseed reference genome (accessed on 10 July 2021 https://ftp.ncbi.nlm.nih.gov/genomes/all/GCF/000/686/985/GCF_000686985.2_Bra_napus_v2.0/GCF_000686985.2_Bra_napus_v2.0_genomic.fna.gz) by TopHat. The transcript abundances of genes were estimated using fragments per kilobase of exon per million fragments mapped (FPKM) using the R package “edgeR” [58]. Additionally, differentially expressed genes (DEGs) were identified using the R package “DESeq”. The threshold determining the significance of DEGs among multiple tests was set at an adjusted *p*-value < 0.05 and |log_2_fold changes| ≥ 1. Gene ontology (GO) and the Kyoto Encyclopedia of Genes and Genomes (KEGG) enrichment analyses of DEGs were performed using a “clusterProfiler” R package [59] and the KOBAS website (accessed on 20 August 2021 http://kobas.cbi.pku.edu.cn/) [60] at a significance threshold of an adjusted *p*-value < 0.005 and a *Corrected p*-value < 0.005, respectively.

### 4.4. ROS content and Antioxidant Enzyme Activity Assay

Samples of approximately 0.1 g were ground in an ice bath by adding 1 mL cold phosphate-buffered saline (PBS; pH 7.8) and 0.02 g of quartz sand. Then, the sample mixture was centrifuged at 12,000 rpm for 20 min at 4 °C to collect the supernatant. The content of H_2_O_2_ and O^2−^ and the antioxidant enzyme activities of superoxide dismutase (SOD) and catalase (CAT) were determined with three biological replicates using a Total Oxidative or Antioxidant Capacity Assay Kit (Sinobestbio, China), following the manufacturer’s instructions. Briefly, the O^2−^ content was detected using a hydroxylamine hydrochloride reduction method [61], and the H_2_O_2_ content was detected with titanous sulfate [62]. Superoxide dismutase (SOD) activity was detected using the nitroblue tetrazolium (NBT) method [63]. CAT activity was determined at 240 nm with pH 7.0 phosphate-buffered saline (PBS) (0.15 mol/L) as a control. A change in absorbance of 0.1 per min was taken as 1 enzyme activity unit (U) [63]. All enzyme activities were measured with a microplate reader.

### 4.5. Quantitative RT-PCR

Reverse transcription of mRNA from the siliques was conducted using the FastQuant RT Super Mix (TIAN-GEN, China). The qRT-PCR amplification of randomly chosen genes was performed using a 2 × SYBR Green qPCR Master Mix (US Everbright^®^Inc., Suzhou, China) on a CFX96 Touch Deep Well^™^ Real-Time PCR Detection System (Bio-Rad, USA) with three biological replications (Table S1). The expression levels of genes were calculated using the 2^−ΔΔCt^ method. The expression level of *BnActin7* was used as an internal control to normalize the transcript level.

## Data Availability

Data are available upon request.

## Supplementary Materials

The following supporting information can be downloaded at: https://www.mdpi.com/article/10.3390/plants12142622/s1, Table S1: List of qRT-PCR primer sequences; Table S2: List of DEGs in AE vs. Bna and NE vs. Bna; Table S3: List of significantly enriched GO terms for DEGs in AE vs. Bna; Table S4: List of significantly enriched KEGG pathways for DEGs in AE vs. Bna; Figure S1: Gene expression of 20 randomly selected DEGs in siliques, as detected via qRT-PCR and RNA-seq at 8 and 16 DAP; Figure S2: Heat map of expression of genes involved in cell growth and growth hormones in AE and NE, compared with Bna, in siliques at 8 and 16 DAP; Figure S3: Determination of the content of O^2−^ and H_2_O_2_ and enzyme activity of superoxide dismutase (SOD) and catalase (CAT) in silique, ovule and pericarp at 8 and 16 DAP in AE and NE.
