# Supplementary material for: Comparative Transcriptome Analysis Points to the Biological Processes of Hybrid Incompatibility between Brassica napus and B. oleracea
Source: Plants (Basel). 2023 Jul 12;12(14):2622. doi: 10.3390/plants12142622 (PMC10384443; doi:10.3390/plants12142622)
Supplement: Supplementary file 1 [file plants-12-02622-s001.zip › Supplementary Figure_hybrid incompatibility.pdf]

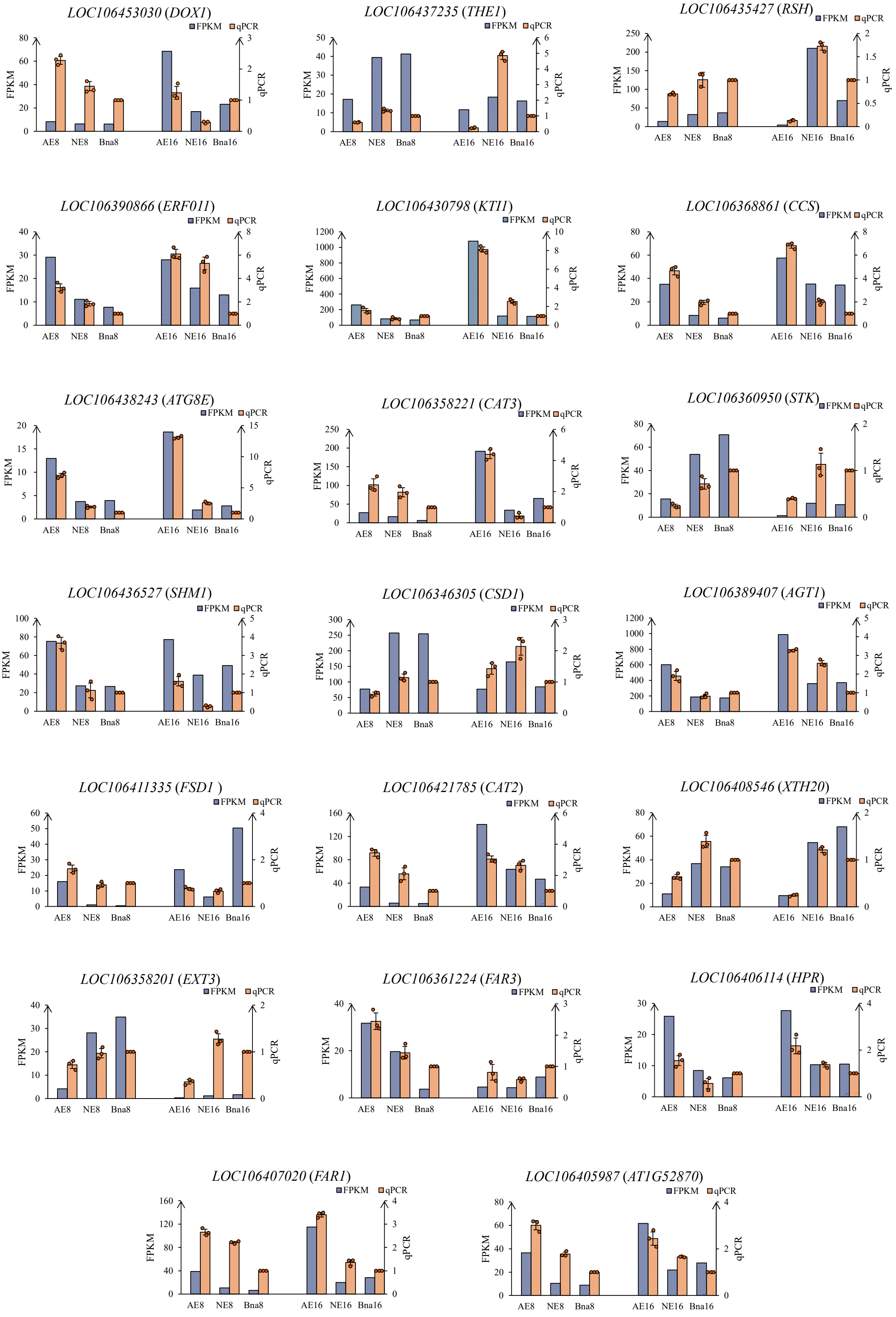


Figure S1. Gene expression of twenty randomly selected DEGs in silique detected by qRT-PCR and RNA-seq at 8 and 16 DAP.

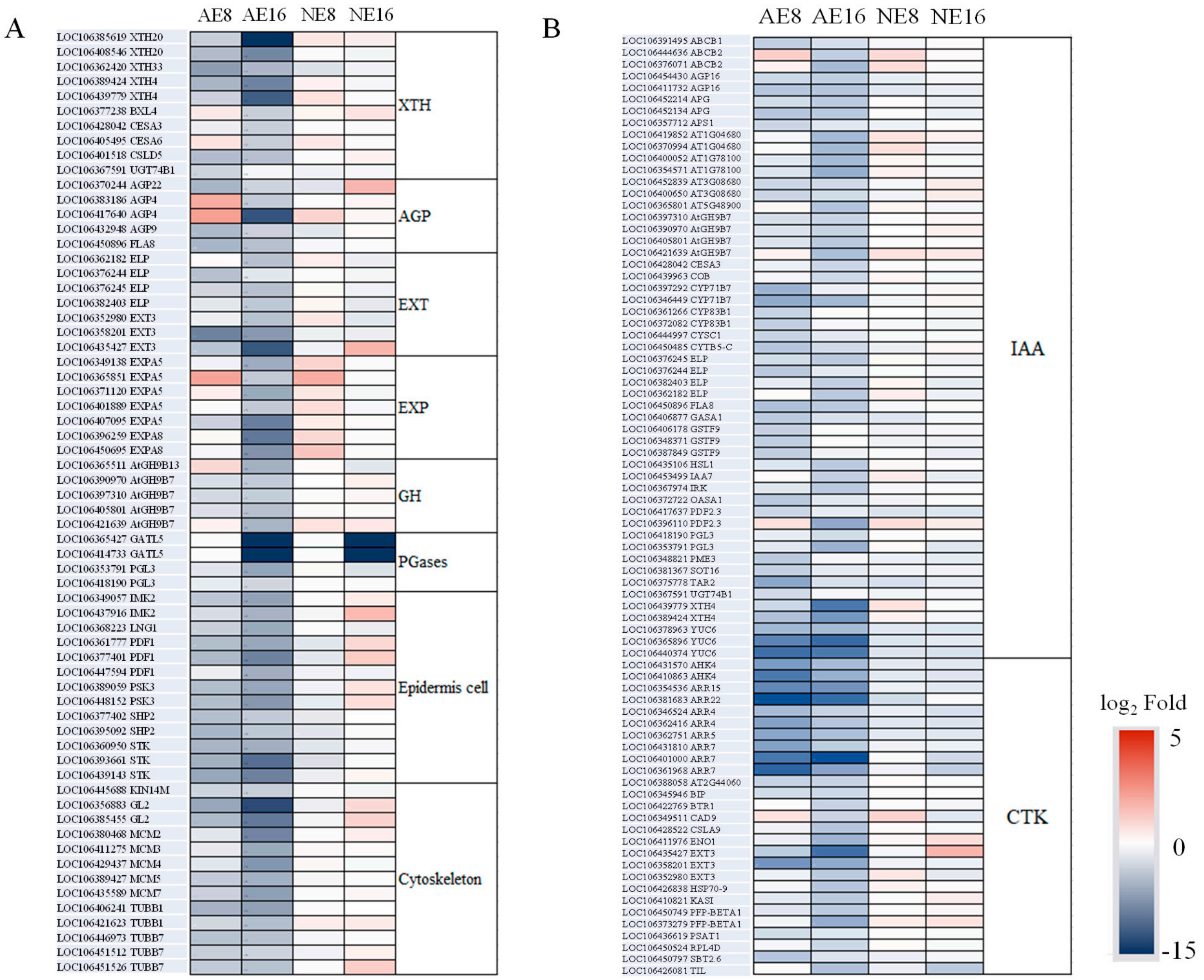

Figure S2. Heat map of genes expression involved in cell growth (A) and growth hormone (B) in AE and NE compared with Bna in silique at 8 and 16 DAP. The color in each cell represents the level of gene expression based on the $\log_2$ fold.

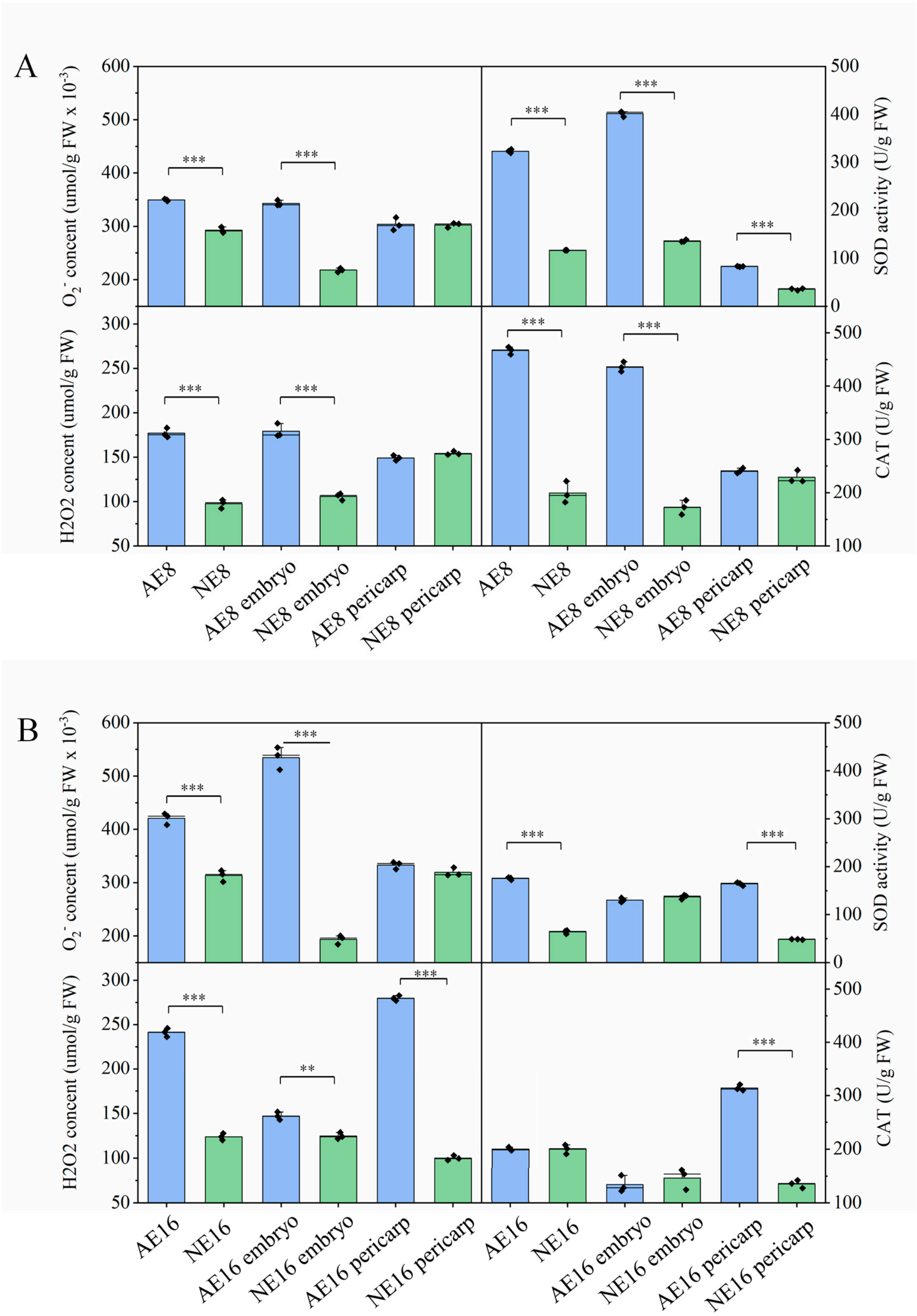


Figure S3. Determination of the content of $O^{2-}$ and $H_2O_2$ and enzyme activity of superoxide dismutase (SOD) and catalase (CAT) in silique, ovule and pericarp at 8 (A) and 16 (B) DAP in AE and NE. *** Significant difference at $p$ <0.001.
